# Supplementary material for: Experimental noise cutoff boosts inferability of transcriptional networks in large-scale gene-deletion studies
Source: Nat Commun. 2018 Jan 9;9:133. doi: 10.1038/s41467-017-02489-x (PMC5760630; doi:10.1038/s41467-017-02489-x)
Supplement: Supplementary file 1 — Supplementary Information [file 41467_2017_2489_MOESM1_ESM.pdf]

Supplementary Note 1

Counting the number of inferable links as a function of perturbed nodes

The expected fraction of inferable links can be calculated analytically if (i) the N_p externally perturbed nodes are uniformly distributed throughout the network, (ii) measurement noise is absent, (iii) additional perturbations – such as internal perturbations arising from thermal noise – are absent, (iv) links are assumed to be represented by noiseless, linear functions with non-zero slope, and (v) an arbitrary large set of perturbation experiments can be generated, with the freedom to tune the perturbation strength for each node independently. Throughout this work we do not assume that networks belong to the class of directed acyclic graphs (DAG), which is often a necessary prerequisite for approaches based on Bayesian networks [1] and structural equations models [2]. The assumption that perturbed nodes are uniformly distributed throughout the network is done for mathematical convenience and can be generalised to other distributions. However, if the distribution of perturbed nodes correlates with features that affect inferability, such as the outdegree, the maximum fraction of inferable links can change significantly. We assume links to be represented by linear functions as otherwise inferability would depend on the functional realisation of each link due to fact that any saturating behaviour by sigmoidal functions can limit the information that can be transmitted, which in turn can affect the fraction of inferable links. Moreover, a linear network model has the important property that independent single-node perturbation experiments can be linearly combined to compute the expected responses of multi-node perturbation experiments.

To count the number of inferable links under the conditions (i)-(v) we assume that for a given subnetwork a perturbed node (A) targets a node (B), given that the outdegree of node A is k, the outdegree of node B is l, and A and B have c nodes as common targets (Supplementary Figure 1). We denote a directed link from source node (A) to target node (B) as inferable if there exits a detectable amount of mutual information between A and B that cannot be transmitted by any alternative route through the network. This requires that at least one node of each alternative route is perturbed and thereby part of the transmitted information is destroyed. It is important to recognise that it is necessary and sufficient to perturb only the shortest alternative routes that pass through the outgoing nodes of A or B, as we assume that all possible network structures – and thus also the shortest ones – occur with the same prior probability and information about A spreading through the network must pass through the outgoing nodes of A or B. To meet

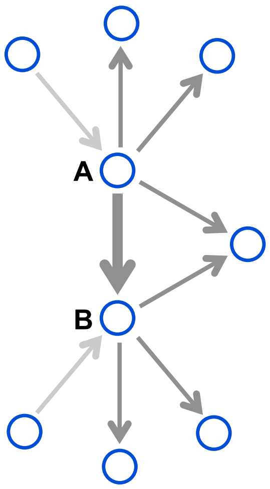

Supplementary Figure 1: Example of a directed subnetwork. We would like to infer the link from node A to node B (shown in bold), where A has outdegree 4, B has outdegree 3, and A and B share 1 common target.

this requirement, either one of the following conditions must be fulfilled (Fig. 2a, main text):

1. All nodes that are targeted by A are perturbed, including node B.
2. Except B, all nodes that are targeted by A and B are perturbed.

By collecting all subnetworks that fulfil conditions 1 or 2 we can calculate the average fraction of inferable links, $F(N_p)$, using $N' := N - k - 1$ and $N_p' := N_p - k - 1$

$$F(N_p) = \sum_k \frac{\binom{N'}{N_p'}}{\binom{N}{N_p}} P(k|A \to B) + \sum_{k,l,c} \frac{\binom{N'-l+c}{N_p'-l+c+1}}{\binom{N}{N_p}} P(k,l,c|A \to B) \quad (1)$$

Here, N is network size, N_p is number of perturbed nodes, k is the outdegree of the source node (A), l is the outdegree of the target node (B), c is the number of common nodes targeted by A and B. We further defined by $P(k|A \to B)$ the conditional probability that for any two connected nodes, source node (A) has

outdegree k and $P(k, l, c|A \to B)$ is conditional probability that for any two connected nodes, source node (A) has outdegree k , target node (B) has outdegree l, and A and B target c common nodes. The first term in the numerator counts motifs that fulfil condition 1, and the second term in the numerator counts motifs that fulfil condition 2. The term in the denominator counts all possible network motifs, when N_p nodes of the network are perturbed.

Assuming that the network size, N, and the number of perturbed nodes, N_p, are much larger than the outdegrees, $N, N_p \gg k, l$, we can apply Stirling's approximation and simplify Eq. (1)

$$F(q) \approx \sum_{k,l,c} \left[q^{k+1} + (1-q)q^{k+l-c}\right] P(k, l, c|A \to B) \tag{2}$$

where q denotes fraction of perturbed nodes, $q = N_p/N$.

Network's Inferability

According to Eq. (2), networks with different structural features have different $F(q)$ curves (Fig. 1b, main text). We therefore define an inferability measure, I_F, as the area under the curve of F(q) that reflects how difficult it is to infer links for a given network structure

$$\begin{aligned} I_F &= \int_0^1 F(q)dq \\ &= \sum_{k,l,c} \left[\frac{1}{k+2} + \frac{1}{k+l-c+1} - \frac{1}{k+l-c+2}\right] P(k, l, c|A \to B) \end{aligned} \tag{3}$$

Note that I_F is independent of network size. For sufficiently large networks $N \gg 1$ and when feed forward loops are rare, we can approximate the joint probability by

$$\begin{aligned} I_F &\approx I_F^* \\ &:= \sum_{k,l,c} \left[\frac{1}{k+2} + \frac{1}{k+l+1} - \frac{1}{k+l+2}\right] P(k|A \to B)P(l|A \to B) \end{aligned} \tag{4}$$

According to Eqs. (3) and (4), inferability mainly depends on the outdegree of nodes. Consequently, networks consisting of nodes with high outdegree have low inferability (I_F) and are the most difficult ones to infer.

Comparison of $F(N_p)$ with the inference algorithm

We compared the fraction of inferable links determined by the analytical formula, $F(N_p)$, and the average number of inferable links classified by our inference algorithm in the limit of low measurement noise. In order to calculate the average number of inferable links from the inference algorithm, we focus on scale-free and random networks. For each number of perturbed nodes, N_p, we first randomly select the nodes that will be perturbed and subsequently generate node activity data for single-node perturbations. For each random sample of N_p nodes, we calculate the fraction of inferable links and finally average over all configurations of perturbed nodes. We compared the results of the analytical and numerical approaches for three different types of networks (Supplementary Figure 2). We considered a scale-free network where hubs are targets of links (Supplementary Figure 2-a), a scale-free network where hubs are sources of links (Supplementary Figure 2-b), and a random network (Supplementary Figure 2-c). For all three network types, the network sizes are $N = 100$ and averaging runs over 300 different configurations of N_p randomly selected nodes that are perturbed individually by knockouts.

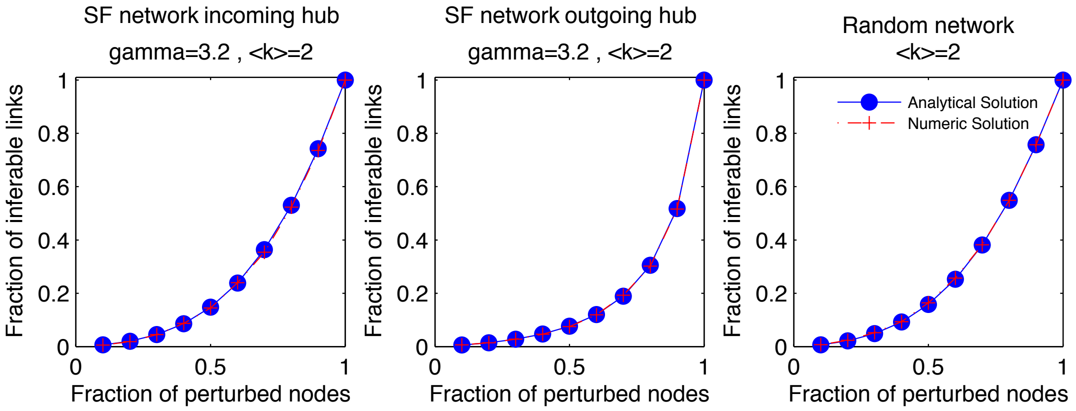

Supplementary Figure 2: Comparison of the analytical formula, Eq. (1) and the inference algorithm by calculating the number of inferable links with respect to the number of perturbed nodes, N_p.

Supplementary Note 2

Interpretation of network inference algorithms that are related to partial correlations

Algorithms based on partial correlations [3, 4] – a method designed to discriminate between direct and indirect interactions – show comparatively low performance for predicting direct correlations between transcript levels using transcriptome data [5] but excellent performance for predicting contact points of protein structures [6, 3]. To give a possible reason for this difference in performance it is illuminating to solve the linear model of Methods Section for the case that (i) the perturbations, $\boldsymbol{u}$, are fast fluctuating, independent, and identically distributed, (ii) the network is undirected, $\boldsymbol{A} = \boldsymbol{A}^T$, and (iii) measurement noise is absent. The resulting time evolution equation is given by

$$\frac{d\boldsymbol{y}(t)}{dt} = \boldsymbol{A}\left(\boldsymbol{y}(t) - \boldsymbol{y}^{ref}\right) + \boldsymbol{u}(t) \quad (5)$$

with solution

$$\boldsymbol{y}(t) = \boldsymbol{y}^{ref} + \int_{-\infty}^{t} e^{\boldsymbol{A}(t-t')}\boldsymbol{u}(t')dt' \quad (6)$$

We further assume that $\boldsymbol{u}(t)$ fluctuates sufficiently fast such that it can be modelled as Gaussian white noise, with $\mathbb{E}[\boldsymbol{u}(t)] = \boldsymbol{0}$ and covariance $\mathbb{E}[\boldsymbol{u}(t)\boldsymbol{u}(t')] = 2\delta(t-t')\boldsymbol{I}$, where we defined with $\delta(t)$ the delta function and by $\boldsymbol{I}$ the identity matrix. As a consequence, the resulting covariance matrix of node activities is given by

$$\boldsymbol{C} := \mathbb{E}\left[(\boldsymbol{y}^{obs} - \boldsymbol{y}^{ref})(\boldsymbol{y}^{obs} - \boldsymbol{y}^{ref})^T\right] = -\boldsymbol{A}^{-1} \qquad \Leftrightarrow \qquad \boldsymbol{A} = -\boldsymbol{C}^{-1} \quad (7)$$

where the expectation is taken over the stochastic variable $\boldsymbol{u}(t)$. This shows that the elements of interaction matrix, $\boldsymbol{A}$, are related to the inverse of the covariance matrix and thus a nonzero element of $\boldsymbol{A}$ implies non-zero partial correlation. As the constraints imposed by the conditions (i)-(iii) do typically not hold for transcriptional networks [5] – where most of data is generated by perturbations that persist on long time scales, measurement noise is significant, and the network is directed – inference of such networks from inverting correlation matrices should be interpreted with care [3, 4]. In contrast, conditions (i)-(iii) are satisfied to very good approximation for identifying physical contacts between amino acids in folded proteins [6], where single nucleotide substitutions occur on much shorter time scales than significant changes in protein structure, links are undirected, and measurement noise from sequencing errors make no relevant contribution.

To see the difference between partial correlations and response matrix approaches – to which our method of partial response coefficients (PRC) belongs – we focus on the special case that slow fluctuating perturbations are applied individually to all nodes in the network and measurement noise is absent [7]. For this special of complete coverage with perturbed nodes, the partial response coefficients can be calculated directly from Eq. (2) of the Methods Section. In this case the response of the i-th node, δy_i, with respect to a perturbation that acts exclusively on the j-th node is given by $\delta y_i = -A^{-1}_{ij} \boldsymbol{B}_j \boldsymbol{u}$, with $\delta y_i := y_i - y_i^{ref}$ and $\boldsymbol{B}_j$ the j-th row of $\boldsymbol{B}$. Using the expression $\delta y_j = -A^{-1}_{jj} \boldsymbol{B}_j \boldsymbol{u}$ to substitute $\boldsymbol{B}_j \boldsymbol{u}$, we arrive at the relation

$$G_{ij} := \frac{\delta y_i}{\delta y_j} = \frac{A^{-1}_{ij}}{A^{-1}_{jj}} \qquad \Rightarrow \qquad \boldsymbol{G} = \boldsymbol{A}^{-1} [diag \boldsymbol{A}^{-1}]^{-1} \tag{8}$$

with $diag \boldsymbol{X}$ a matrix with elements $\{X_{11}, X_{22}, ...\}$ on the main diagonal and zero elements otherwise. Here, $\boldsymbol{G}_{ij}$ denotes the linear response coefficient of the network, describing the change in activity of node i if node j changes its value in response to a perturbation that acts exclusively on node j. After inverting both sides of Eq. (8) and setting the restoring forces (degradation rates) to unity, $A_{jj} = -1$ for all j, we arrive at the central result of Ref. [7]

$$\boldsymbol{A} = [diag \boldsymbol{A}^{-1}]^{-1} \boldsymbol{G}^{-1} = -[diag\, \boldsymbol{G}^{-1}]^{-1} \boldsymbol{G}^{-1} \quad . \tag{9}$$

In the derivation we used that by definition $diag \boldsymbol{A} = -\boldsymbol{I} = [diag \boldsymbol{A}^{-1}]^{-1} diag\, \boldsymbol{G}^{-1}$, with $\boldsymbol{I}$ the identity matrix.
The lack of a unique relation between $\boldsymbol{G}$ and $\boldsymbol{A}$ is rooted in the fact that the $N(N+1)$ parameters of $\boldsymbol{A}$ and $\boldsymbol{Bu}$ cannot be uniquely identified from the $N \times N$ measured node activities, even for the rare case that all nodes in the network are perturbed one-by-one. This problem does not change if more perturbations are carried out as $\boldsymbol{Bu}$ takes different values for different perturbations. The result that direct interactions can be computed by inverting the linear response matrix, Eq. (9), has been derived several times, with either fixing the restoring force, $diag \boldsymbol{A} = -\boldsymbol{I}$, [7, 4] or by computing direct interactions from $\boldsymbol{A} = \boldsymbol{I} - \boldsymbol{G}^{-1}$, [3] (the latter reference defines an unusual response matrix with zero elements on the main diagonal which in their notation reads $\boldsymbol{G}_{obs} := \boldsymbol{G} - \boldsymbol{I}$).

Substantial confusion arises if $\boldsymbol{G}$ is substituted by $\boldsymbol{C}$ in Eq. (9) for the reason that $\boldsymbol{C}$ can be computed using perturbative forces that act simultaneously on many nodes [4, 3] and knowledge which nodes are perturbed in an experiment is not required, whereas computation of $\boldsymbol{G}$ requires that all nodes are perturbed individually and the identity of the perturbed nodes needs to be known [7]. However, the substitution $\boldsymbol{G} \to \boldsymbol{C}$ in Eq. (9) is in general invalid, and leads only to meaningful

interaction matrix $\boldsymbol{A}$ if the conditions (i)-(iii) apply [5].

Supplementary Note 3

Input data to network inference algorithm

Saccharomyces cerevisiae genome-wide knockout library

Description of the data set

Kemmeren et al. [8] provided a transcriptome data set of a *Saccharomyces cerevisiae* genome-wide knockout library (with mutant strains isogenic to S288c [9]). This data set, hereafter simply referred to as the yeast deletome data, comprises of transcript levels of 6170 genes for 1484 deletion mutants (hereafter referred to as perturbation experiments). The data of 1441 of these perturbation experiments can be used for network inference as the transcript levels of the deleted genes was measured with the microarray chip design. The data is presented as the logarithm of the fluorescence intensity ratios of red (R) and green (G) labelled microarray targets, referred to as M-values: $M = \left(\frac{R}{G}\right)$. For the regular experimental setup, DNA from the deletion mutants was labelled red, and DNA that was pooled from several wild type batches was labelled green. The aim of pooling the wild type DNA was to average out biological fluctuations, so that this DNA pool could be used to define a reference gene expression level. This allows the interpretation of the M-values as logarithmic fold changes of mutant gene expression levels compared to a wild type reference. Kemmeren et al. also used a dye swap setup for several experiments to average out the effect of a possible dye bias. The chip design used by Kemmeren et al. measures each gene twice per biological sample, thus allowing estimation of the technical variance.

Preprocessing of data

We used the preprocessed data (M-values) provided by Kemmeren et al. for our analyses. The preprocessing steps are described elsewhere [8]. We re-arranged the original preprocessed data layout to make it compatible with our network inference algorithm. Specifically, M-values corresponding to dye-swap experiments were multiplied by -1 and only knock-out experiments corresponding to genes that were also included in the chip design were kept in the data set.

Distribution of biological and technical variance (figure 3a and figure 3b)

Since there is insufficient information about the biological processes leading to variation among biological replicates (biological noise), we assume that the process is equal for all genes. By bringing the wild type data to unit variance for each gene, we show that the variation among the wild type data logarithmic fold changes is approximately Gaussian distributed (figure 3a). By taking the mean logarithmic fold change of each biological replicate (wild type data only) and then calculating the differences of the technical replicates to this mean, the distribution of the technical variance can be found (figure 3b).

Correlations among wild type experiment M-values (figure 3c)

The histograms shown in figure 3c were created from the upper triangular correlation matrix (without diagonal elements) of wild type experiment M-values. The inserted figure was created by breaking up correlations between genes via random shuffling of the experiments.

Simulated Gene Expression data

To estimate how well network inference works on data which is similar to the data set provided by Kemmeren et al. [8], the input data for the simulations was generated as described below. In short, steady state solutions of ordinary differential equations were used to generate absolute gene expression levels, which were then turned into logarithmic fold changes, to which measurement noise was added.

Using scale free networks and steady state solutions of ordinary differential equations to simulate absolute gene expression levels

We chose scale free network models with exponent 2.5 to simulate a comparable network structure to yeast; the average degree was set to 2. Of all links, 80 % were set as inhibiting. Non-linear regulatory interactions were simulated by solving ordinary differential equations. First, wild type expression levels were calculated by solving a system of equations of the form shown below. The Hill-coefficients h_{ki} were sampled from a uniform distribution between 1 and 2. The constant K_{ki} was set to 0.5. The linear degradation term λ_i was set to 2 to assure stability. The coefficients a_{ki} and b_{ki} were set to 1 and -1 respectively for inhibiting links and to 0 and 1 respectively for activating links.

$$\dot{y}_i = \sum_{k\ \in \{1,\ \ldots,\ N\}/i} \left(a_{\mathrm{ki}} + b_{\mathrm{ki}} \frac{y_k^{h_{\mathrm{ki}}}}{K_{\mathrm{ki}}^{h_{\mathrm{ki}}} +\ y_k^{h_{\mathrm{ki}}}} \right) +\ u_i -\ \lambda_i y_i$$

Here, u_i denotes the basal gene expression rate. To compute the response y_{ij} of a gene i to a knock-out of gene j, the above shown system of ODEs was modified by setting the basal expression rate for the knocked out gene to zero and by removing all links onto and away from the gene j.

Using GeneNetWeaver to simulate absolute gene expression data

For better comparability with other publications, gene expression data was also generated with the program "GeneNetWeaver" [10]. This data was only used for comparing inference methods using ROC curves. From the "gold standard" yeast network in the program, random subnetworks were extracted with at least 100 regulators (random vertex seed; greedy neighbor selection). Then, the "Generate Kinetic Model" option was used with removal of auto-regulatory interactions to allow the generation of data sets. Data sets were generated with the following options: deterministic (ODEs) model, knock-out & wild type experiments, no time series, no noise added.

M-values (Logarithmic fold changes)

To simulate logarithmic fold changes that were similar to the M-values (logarithmic fold changes) of the yeast deletome data set described above, the M-values of yeast knock-out mutants were investigated for the knocked out genes. Due to measurement noise, these values never reach negative infinity as would be expected in the absence of technical noise. The median minimal absolute fluorescence intensity averaged over all knocked out genes was estimated to be $2^{-2.5}$. This value was added to all simulated absolute gene expression level values before calculating the logarithmic fold changes. That is, the gene expression response of a gene i towards a perturbation of a gene j, expressed as logarithmic fold change, was calculated as $M_{ji} = log_2\left(\frac{y_{ji}+2^{-2.5}}{y_i+2^{-2.5}}\right)$, with y_{ji} the absolute expression level of gene i when gene j is perturbed, and y_i the expression level of gene i for the non-perturbed (wild type) state, as defined above. Replicates were produced through duplication of the logarithmic fold changes for each perturbation experiment.

Simulating measurement noise

Although the reference node activities in the data set provided by Kemmeren et al. [8] are correlated, we did not simulate correlated reference node activities.

This is because we were lacking information about the processes that generate biological variance as well as the actual network structure of *S. cerevisiae*. Because the biological noise dominated the technical noise, we did not distinguish both noise types in our simulations. Rather, we simulated noise by adding gaussian distributed random numbers to all simulated M-values.

DREAM3 in silico challenge data

As explained in more detail elsewhere [11], participants were given 3 data sets called "null-mutant", "heterozygous" and "trajectories" for each network. The "null-mutant" and "heterozygous" data sets contained wild type gene expression levels as well as the steady state gene expression levels of single gene knock-outs for all genes. In both cases, it was indicated which data belonged to which knock-out (perturbation target). The "trajectories" data sets contained several time-series of gene expression but did not contain information about the perturbation target. We used the "null-mutant" data sets as well as the near-stationary part ($t \geq 170$) of the trajectories data. The former was used to center the gene expression levels (node activities) and to calculate the covariance matrix. The latter was used to estimate the overall noise level.

Workflow

In the following, work flow steps are listed in the order they were possibly used. Depending on the analysis, not all of the steps were used. For example, for the analysis of DREAM3 data, the steps for clustering and for the identification of significant links were skipped.

Data centering

For all analyses (yeast deletome, simulations & DREAM3 challenge), the node activity data was centered to the average of the corresponding reference node activities (not to the average of all node activities).

Identification of significantly affected nodes

To identify which nodes were significantly affected by a perturbation, it was checked whether the node activities were significantly different from the background noise. This was done by comparing node activities magnitude of perturbation experiments to the corresponding reference node activity variance using two-tailed t-tests (yeast deletome data & simulated data) or two-tailed z-tests (DREAM3 challenge data).

Clustering

Nodes were grouped into clusters if they were not sufficiently linearly independent. The following steps were followed:

1. All node activities were normalized to unit reference node activity variance.

2. A $(P \times N)$ node activity matrix was formed by merging experimental replicates (here, P denotes the number of experiments and N denotes the number of genes). Replicates (biological and technical) were merged by averaging and multiplication by the square root of the number of biological replicates.

3. For each pair of nodes k and l, a test statistic T_{kl} was compared to a 5 % FDR cutoff. The test statistic was calculated as the square of the smaller singular value of the $P \times 2$ matrix consisting of those two columns of the node activity matrix which corresponded to the nodes k and l. For simplicity, we assumed that the reference node activities were Gaussian distributed and that the node interactions were sufficiently linear. Then, under the Null Hypothesis of linear dependence, the test statistic follows a distribution which we approximated with a Chi-square distribution with P degrees of freedom (the expected value of the $\mathcal{X}_P^2$ distribution over-estimates the expected value of the small eigenvalue, but this is negligible if sufficiently high confidence is claimed to reject the null hypothesis). An FDR of 5 % was chosen to define a significance cutoff.

4. Clusters were finally formed by grouping unperturbed nodes with perturbed nodes on which they were linearly dependent. If an unperturbed node was linearly dependent on multiple perturbed nodes, this node was grouped with the perturbed node onto which it was the most linearly dependent. Remaining unperturbed nodes were grouped with other unperturbed nodes if they were not sufficiently linearly independent.

5. For each cluster, either the perturbed node, or, if no perturbed node existed in the cluster, the node with the largest signal-to-noise ratio was chosen to be the cluster representative, and the corresponding node activity data was used for further analysis.

Links between clusters were found by inferring links between cluster representatives. Links within clusters can only be inferred within two-node clusters if there is exactly one perturbed node and if the unperturbed node is the only node affected by the perturbed node. Note that all perturbation experiment node activities were used to create the $(P \times N)$ node activity matrix and that no bootstrapping was done.

Subnetwork method

To infer a link from a node j onto a target node i using the subnetwork method, the network is temporarily limited to only those nodes that are significantly affected when node j is perturbed (the cutoffs used here were the same ones that were used for identifying the significantly affected nodes). This means that the inference of several links is skipped, and the corresponding link strengths are set to zero and are not included in the calculation of the FDR to determine a cutoff for link significance.

Identification of significant links

Partial Response Coefficients (PRCs) were calculated for the respective subnetworks. Then, inferable links were tested for significance using z-tests. The link strength's variances were estimated by bootstrapping over the node activity data (described below). The false discovery rate (FDR) was adjusted to 5 % to account for multiple hypothesis testing [12].

Bootstrapping

Residual bootstrapping was used (this was done to get a speed-up compared to parametric bootstrapping, in which residuals are drawn de novo from a distribution rather than used multiple times but in randomized order). First, residuals were calculated from the reference node activity data and then added to the average node activity values of each perturbation experiment. Depending on the analysis, we followed one of two separate approaches for calculating the residuals from the data for reasons described in the following.
First, because we had found that wild type experiment logarithmic fold changes are highly correlated in the yeast deletome data, we sought to maintain the correlations among the reference node activity residuals to improve inference performance.
Second, because we did not simulate correlated measurement noise in our simulations, the effect of random correlations among the reference node activities resulting from limited sample sizes was sought to be minimized. This was achieved by shuffling the the order of reference experiments among the nodes.
Both procedures are explained in the following section.

Residual bootstrapping procedure illustrated

Both bootstrapping approaches are illustrated in the following example, in which it is described how residual bootstrapping is performed on the node activity data for one perturbation experiment. (Residual bootstrapping of reference node activity data is analogous). The node activity data for N reference experiments of

a two-node network with nodes i and j is represented as column vectors $\mathbf{x}_i^{WT} = \left(x_{i,1}^{WT},\ \ldots,\ x_{i,N}^{WT}\right)^T$ and $\mathbf{x}_j^{WT} = \left(x_{j,1}^{WT},\ \ldots,\ x_{j,N}^{WT}\right)^T$, and node activity data for a single perturbation experiment with two replicates is represented as $\mathbf{x}_i^{KO} = \left(x_{i,1}^{KO},\ x_{i,2}^{KO}\right)^T$and $\mathbf{x}_j^{KO} = \left(x_{j,1}^{KO},\ x_{j,2}^{KO}\right)^T$. The residuals for the nodes are calculated to be $\mathbf{r}_i{=}(r_{i,1},\ldots,r_{i,N})^T = \bar{x}_i^{WT} - \mathbf{x}_i^{WT}$ and $\mathbf{r}_j{=}(r_{j,1},\ldots,r_{j,N})^T = \bar{x}_j^{WT} - \mathbf{x}_j^{WT}$, with $\bar{x}_j^{WT}$ and $\bar{x}_i^{WT}$ the mean of the corresponding reference node activities. To leave correlations among reference node activities intact, we have the following expression for an arbitrary bootstrap sample for the perturbation node activities: $\mathbf{x}_i^{KO,boot} = \left(\bar{x}_i^{KO}, \bar{x}_i^{KO}\right)^T - (r_{i,p}, r_{i,q})^T$ and $\mathbf{x}_j^{KO,boot} = \left(\bar{x}_j^{KO},\ \bar{x}_j^{KO}\right)^T - (r_{j,p}, r_{j,q})^T$, with p and q being random (possibly equal) integers between 1 and N, and $\bar{x}_i^{KO}$and $\bar{x}_j^{KO}$the average node activities of the perturbation experiment. To destroy correlations among reference node activity residuals, the residuals are shuffled randomly across bootstrap samples. We have the following expression for an arbitrary bootstrap sample for the perturbation node activities: $\mathbf{x}_i^{KO,boot} = \left(\bar{x}_i^{KO}, \bar{x}_i^{KO}\right)^T - (r_{i,p}, r_{i,q})^T$ and $\mathbf{x}_j^{KO,boot} = \left(\bar{x}_j^{KO},\ \bar{x}_j^{KO}\right)^T - (r_{j,u}, r_{j,v})^T$, with $p,\ q, u$ and v being random (possibly equal) integers between 1 and N.

Applications

Analysis of the yeast deletome data

Work flow steps for the Inference of the GAL network

The network was limited to all genes that could in principle be affected by perturbations of the following nodes: GAL3, GAL4, GAL80 and MIG1. A false discovery rate of 10^{-3} was used as a threshold for the identification of significantly affected nodes. With this threshold, 44 nodes remained in the network, that were then clustered. Then, subnetworks were determined, followed by inference of links between clusters. Only clusters that contained at least one of the above mentioned nodes were kept for the graph shown in figure 4d.

Note on the identification of significantly affected nodes

Because we had found that, among the node activities, the biological variance was larger than the technical noise, only the number of biological replicates was used to estimate the degrees of freedom. To account for the multiple hypothesis testing, we adjusted the overall false detection rate (FDR) [12]. For the analysis of the *Saccharomyces cerevisiae* genome-wide knockout library, we followed the recommendation by Kemmeren et al. [8] (supplementing information) to exclude several genes from further analysis. Furthermore, we excluded all Pseudogenes

and dubious ORFs listed on yeastgenome.org [13]. Because we had found that the distribution of normalized wild type experiment logarithmic fold changes can be well described by a t-distribution with approximately 11 degrees of freedom, this number was used as the degrees of freedom for the t-tests instead of the number of biological wild type experiments. Although the microarray design used by Kemmeren et al. [8] could be used to disentangle technical and biological noise, the biological noise clearly dominated over the technical noise. Hence, we did not disentangle the variances for our analyses but rather used the overall variance. Nodes that were significantly affected in at least one perturbation experiment were kept in the network; all other nodes were removed from further analysis.

Lists of inferred links (Supplementary data 1 and 2)

To generate a list of the most significant links inferred from the whole yeast deletome data, a consensus list was created that reflects the links inferred from the data by using two different methods of bootstrapping (see also the section on bootstrapping). The reason for this is that we are lacking a sophisticated model describing the process that generates biological noise. A consensus list should thus represent a more reliable model for link inference, as only the most significant links that were found by all methods survive the selection process. We created two different consensus lists (suppl_data1_consensus_stringent.xlsx and suppl_data2_consensus_moderate.xlsx), each corresponding to a certain cutoff to select significantly affected nodes. That is, for the stringent cutoff, the network size was smaller than for the moderate cutoff because less nodes were found to be significantly affected by perturbations. The cutoffs correspond to false discovery rates of 10^{-8} and 10^{-6}. The lists were merged by keeping only links that appeared in both lists and by then keeping the (at most 500) most significant links with the highest average Z-score. Gene descriptions from the Saccharomyces Genome Database [13] were added to the list to allow easier investigation. We found that, among the most significant links, there are links whose source and target nodes are either adjacent on the genome or which are paralogs. These links are most likely false positives that reflect inspecificities of the gene deletion process used to generate the yeast deletion collection [9].

List of inferred hub nodes (Supplementary data 3)

From the consensus link list with the stringent cutoff for the selection of significantly affected nodes, we selected the 10 nodes with the highest number of significant outgoing links (significance cutoff: 5 % false discovery rate). The mean and median link strengths of the outgoing links were calculated for each of these 10 hub nodes. Gene descriptions from the Saccharomyces Genome Database [13]

were added.

Simulations

Effect of noise-induced bias on relative link strength (figure 3d)

To show that network inference methods are biased towards measurement noise, we simulated node activity data for the case of infinitely many replicates. In that case, all parameters are estimated perfectly because uncertainty resulting from a limited number of observations are averaged out. The covariance matrix of node activities was calculated according to the following formula:

$$\mathrm{C}_{\mathrm{exact}} = \left(A^T\right)^{-1}\left(B^T B\right)(A)^{-1} + \sigma^2 I_N.$$

Where B is a diagonal matrix with element $b_{\mathrm{ii}} = 1$ if node i is perturbed. The signal-to-noise ratio was adjusted by changing the value of σ^2.

Comparison of simple, clustering and subnetwork methods (figure 4c)

The Inferability curves represent averages over 4 different networks and 12 perturbation samples. The network size was 60 nodes. Gene expression data was simulated from scale free networks and a non-linear model (as described below).

Receiver Operating Characteristic (ROC) curves (figure 4b)

Simulation setup The ROC curves were averaged (as described in the paragraph below) over 24 perturbation samples and 4 network structures. The network size was 300 nodes. In each perturbation sample, 25 % of nodes were randomly selected and perturbed individually. The noise-to-signal ratio was adjusted to 10 %. Data was generated with both GeneNetWeaver [10] and scale-free networks with a non-linear model to generate stationary data. For each perturbation sample, only data corresponding to significantly perturbed nodes was used to calculate the covariance matrices (1 % FDR), the only exception being Lasso regression applied to the data of all nodes. All methods (except Lasso applied to the data of all nodes) received almost the same covariance matrix C as input: because of the randomness of bootstrap sampling, small numerical differences between the covariance matrices that PRC and the other methods received may have occurred. Binary classification of inferred links was done solely based on links pointing away from perturbed nodes because only those links are potentially inferable. This is similar to the method described by Siegenthaler and Gunawan [14]. To infer a link onto a certain node, the data corresponding to a perturbation of that node was removed prior to calculating the covariance matrix. Because both Lasso regression

and the subnetwork method set certain link strengths to exactly zero, it would be impossible obtain a ROC curve that smoothly approaches a false positive rate of 1. This is why we set all link test statistics that were equal to zero to random values smaller than the smallest non-zero link test statistic. When creating the ROC curve, this procedure corresponds to random guessing for all links that were set to exactly zero.

ROC curve averaging In the following, the false positive rate is denoted by FPR and the true positive rate is denoted by TPR. To generate an averaged ROC curve, the tuples (FPR, TPR) of the individual ROC curves from each inferred network were assigned to 50 bins according to the FPR of each tuple. Bins were filled up in a way such that approximately equally many tuples were assigned to all bins. Then, for each bin, the FPR and TPR was calculated. These 50 averaged tuples (FPR, TPR) were used for the averaged ROC curve.

ROC curves for Subnetwork method and Lasso We generated 10 different ROC curves for the subnetwork method, each curve corresponding to a different cutoff for the selection of significantly affected nodes (based on which the subnetworks were created). For Lasso, we generated ROC curves for 10 different regularization coefficients. To model a smooth transition between the cutoffs or regularization coefficients, we interpolated (cubic spline) between the ROC curves in a way such that the resulting hybrid curve had a possibly larger area under the curve than the individual ROC curves. The 10 different cutoffs for selection of significant nodes correspond to the following FDRs: 1.00, 0.89, 0.78, 0.67, 0.56, 0.45, 0.34, 0.23, 0.12, and 0.01. The 10 different regularization coefficients were: 0, 0.001, 0.0018, 0.0032, 0.0056, 0.010, 0.0178, 0.0316, 0.0562, and 0.1000.

Comparison of non-regularized inference methods Test-statistics T_{ji} corresponding to a link from a node j to a node i were calculated according to the following formulas. The standard deviations of the test statistics were estimated via residual bootstrap.

1. Regression: $T_{\mathrm{ji}} = \frac{|G_{\mathrm{ji}}|}{\mathrm{std}(G_{\mathrm{ji}})}$, where $G_{\mathrm{ji}} = \frac{(C)^{-1}_{\mathrm{ji}}}{(C)^{-1}_{\mathrm{ii}}}$.

2. Conditional Mutual Information: $T_{\mathrm{ji}} = \frac{|G_{\mathrm{ji}}|}{\mathrm{std}(G_{\mathrm{ji}})}$, with $G_{\mathrm{ji}} = \log_2\left(\frac{\det(A) \times \det(B)}{\det(C) \times \det(D)}\right)$. Here, A, B and D are covariance matrices with rows and columns of certain nodes removed. A: node j removed. B: node i removed. D: nodes i and j removed.

3. PRC: $T_{\text{ji}} = \frac{|G_{\text{ji}}|}{\text{std}(G_{\text{ji}})}$, where $G_{\text{ji}} = \frac{\sum_{k=1}^{N_0} U'_{\text{ik}} U'_{\text{jk}}}{\sum_{k=1}^{N_0} U'_{\text{ik}} U'_{\text{ik}}}$, where N_0 is the number of unperturbed nodes and U_i is the ith eigenvector of the covariance matrix. Note that links were not identified as artifacts.

Comparison of regularized inference methods For a fair comparison, we used 10 different regularization coefficients to generate ROC curves for Lasso regression, keeping only the ROC curve corresponding to the regularization coefficient that gave the best performance. The following is a brief derivation of the formulas used for regularized regression using the L_1 and L_2 norms. The simulation setup is described afterwards. Consider the following equation, which is essentially equation 1 of the main paper except that perturbations are not restricted to single nodes, which is expressed through the perturbation strength matrix U:

$$
\begin{aligned}
\dot{Y} &= YA^T + U \\
Y^{\text{obs}} &= Y + \epsilon
\end{aligned}
$$

For simplicity, we assumed here that the reference state is 0. Here, $Y$are the true node activities that are masked by measurement noise ϵ to yield observed node activities Y^{obs}, and the matrix A^Tdenotes the link strengths. In the steady state and under the assumption that the measurement uncertainties ϵ are Gaussian distributed, a maximum a posteriori (MAP) function of the parameters A^T and U given the observed data Y^{obs} can be defined. The logarithm of this function is:

$$
a\left(A^T, U | Y^{\text{obs}}\right) = K + \sum_{i=1}^{N} \sum_{n \in S_i} \left[\left(\sum_{j=1}^{N} Y_{\text{nj}}^{\text{obs}} A_{\text{ji}}^T \right) + U_{\text{ni}} \right]^2 \frac{1}{\sigma_{\text{ii}}^2} \tag{10}
$$

$$
+ r^2 \sum_{i}^{N} \left(A_{\text{ii}}^T + \mu_i\right)^2 + v^2 \sum_{i}^{N} \sum_{n \in T} U_{\text{ni}}^2 \tag{11}
$$

Here, K is a normalization constant from the distributions that vanishes upon differentiation. The experiment indices n run over the set of all experiments except the ones in which node i is perturbed, S_i. N is the number of nodes; P is the number of experiments; $Y_{\text{nj}}^{\text{obs}}$ is the matrix of node activities (each row corresponds to a sample and each column corresponds to a node); σ_{ii}^2 is a variance coefficient and r^2, v^2 are regularization coefficients that correspond to weights of the prior distributions. Note that the first prior distribution is only defined over the diagonal elements of the network matrix A. It can be shown that the following estimator maximizes the MAP function:

$$
\left(\hat{A}^T\right)_{\text{ji}} = \frac{\left(\Phi^T \Phi\right)_{\text{ji}}^{-1}}{\left(\Phi^T \Phi\right)_{\text{ii}}^{-1}} \mu_i.
$$

Here, Φ denotes the observed node activities with samples corresponding to a perturbation of node i removed (the number of rows of Φ is less than or equal to the number of rows of Y^{obs}, depending on whether node i is perturbed). This means that the data may need to be re-organized to infer links onto each node. To arrive at this result, one needs to maximize $a\left(A^T, U|Y^{\text{obs}}\right)$ with respect to A. This is possible because the diagonal elements of the network matrix A are regularized, which does not allow for arbitrary combinations of either A or U. The following steps lead to the above stated solution:

1. Set the derivative of the log-MAP function w.r.t. U_{ni} to zero: $\frac{\delta a\left(A^T, U|Y^{\text{obs}}\right)}{\delta U_{\text{ni}}} = 0.$

2. Solve for U_{ni} to obtain the MAP estimator $\hat{U}_{\text{ni}}$. Plug this expression into formula for log-MAP function.

3. Set this new expression of the log-MAP function w.r.t. $A^T_{\text{ji}}$to zero: $\frac{\delta a\left(A^T, U|Y^{\text{obs}}\right)}{\delta A^T_{\text{ji}}} = 0.$

4. Solve for A^T_{ji} to obtain the MAP estimator$\hat{A}^T_{\text{ji}}$. To achieve this, one has to apply the Sherman-Morrison-Woodbury formula and let the regularization coefficient r^2 go to infinity, which essentially sets all A^T_{ii} equal μ_i.

Note that, for $\mu_i = -1$, the set of parameters A^T_{ji} (except for A^T_{ii}, which become equal to μ_i) are simply the ordinary least squares (OLS) estimators that minimize the following expression:

$$F = \|\Omega\beta - \Phi_i\ \|_2^2$$

Here, Ω is a matrix that is equal to Φ except that it is missing column i, and β is a vector that is equal to A^T_i except that it is missing the element equal to A^T_{ii}. The L_1 Norm ("Lasso" regularization) and L_2 Norm ("Tikhonov-Miller" regularization) impose further constraints on this extreme value problem. The corresponding formulas are:

$$F_{L1} = \|\Omega\beta - \Phi_i\ \|_2^2\ +\ \gamma_{L_1} \left\|A^T_i\right\|_1$$

and

$$F_{L2} = \|\Omega\beta - \Phi_i\ \|_2^2\ +\ \gamma_{L_2} \left\|A^T_i\right\|_2^2$$

for the L_1 and L_2 Norm, respectively.

DREAM3 in silico challenge

Brief description of the challenge

In the DREAM3 in silico challenge, participants were given the task to infer causal interactions from simulated gene expression data. The challenge was split into 3 sub-challenges, corresponding to the inference of networks with 10, 50 and 100 nodes. In each sub-challenge, 5 networks had to be inferred. Each of these networks had been created by sampling from the set of already known gene regulatory interactions from either *E. coli* or yeast using the tool GeneNetWeaver [10], which had also been used to simulate gene expression data. For each of the 5 networks, the p-values of AUROC and AUPR values had to be estimated, yielding 10 p-values for each sub-challenge. Then, the average of the negative logarithms (base 10) of the p-values was used as the overall score for each sub-challenge, which resulted in one score each for the 10, 50 and 100 node network sub-challenge.

Work flow steps to obtain Partial Response Coefficients (PRC)

After centering the node activity data, significantly affected nodes were determined to obtain subnetworks. The significance level to determine which nodes were significantly affected by a perturbation was adjusted to the network size via Bonferroni correction.

Due to a lack of experimental replicates in the data, we did not perform residual bootstrapping; instead, the raw partial response coefficients (PRC) were used for the inferred network. Furthermore, significant links did not need to be identified since only AUROC and AUPR values had to be calculated.

Calculation of DREAM3 overall scores

We followed the original procedure of the DREAM3 organizers [11], with one modification. The original procedure assumes that a significance measure has been assigned to each inferred link. Because our subnetwork method sets some PRCs to exactly zero (insignificant links), the resulting AUROC and AUPR curves would not be as smooth; furthermore, we observed that the distribution of background AUROC and AUPR values became multimodal for the 10-node network. To circumvent this, we first took the absolute value of all PRCs to obtain a link significance measure. Then, we set all zero PRCs to values smaller than the smallest non-zero link significance measure. Unfortunately, this introduces a randomness in the inferred network that can substantially influence the AUROC value. This is because the AUROC measure is sensitive to links that purely reflect noise. We averaged out this randomness by sampling over the random values that were used to replace the zeros. To obtain p-values for AUROC and AUPR values, we fol-

lowed the original procedure as correctly described in [15], and fitted parametric probability distributions to 100,000 background AUROC and AUPR values. Those background AUROC and AUPR values were obtained by comparing the inferred network to randomly shuffled versions of the reference (gold standard) networks. The DREAM3 organizers used two distribution functions to fit the left and right side of the unimodal background AUROC and AUPR distributions separately. We also investigated the effect of using a t-distribution to fit the AUROC background values, with which we obtained similar p-values.

Supplementary data 4: DREAM3 in silico network inference challenge results

The table (suppl_data4_dream3_scores.xlsx) contains the overall scores as well as AUROC and AUPR values for all networks of each sub-challenge. The first column shows the significance level for significantly affected nodes before Bonferroni-correction. The second column shows the scores when the original method [15] was used for fitting distributions to background AUROC and AUPR values. The third column shows the scores when the original method [15] was used for fitting distributions to the background AUPR values but a t-distribution was used to fit the background AUROC values.

Remarks on Table 1

Table 1 shows the DREAM3 scores from Supplementary data 4 that correspond to a significance level of $\alpha = 0.05$ for the identification of significantly affected nodes (before Bonferroni correction). It includes both the scores that were calculated with the original method as well as with a t-distribution to fit the AUROC background values (described above).
For the 100 node network, it could not be determined whether our approach scored 1st or 2nd. The reason for this is that the score for the highest performing participant in the DREAM3 network inference challenge was published as infinity, as “the p-value for this performance was below the precision” of the calculation of the DREAM3 organizers [11].

Software

For the analyses of the yeast deletome and simulations, we used MATLAB and Statistics Toolbox and Parallel Computing Toolbox Release R2014b, The MathWorks, Inc., Natick, Massachusetts, United States. For the analysis of the DREAM3 challenge data, we used Python 2.7 with numpy 1.11.0, scipy 0.17.0 and mpmath 0.19.

References

[1] Friedman, N. Inferring cellular networks using probabilistic graphical models. *Science* **303**, 799–805 (2004).

[2] Perl, J. *Causality* (Cambridge University Press, 2009).

[3] Feizi, S., Marbach, D., Médard, M. & Kellis, M. Network deconvolution as a general method to distinguish direct dependencies in networks. *Nature Biotechnol.* **31**, 726–733 (2013).

[4] Barzel, B. & Barabási, A.-L. Network link prediction by global silencing of indirect correlations. *Nature Biotechnol.* **31**, 720–725 (2013).

[5] Bastiaens, P. *et al.* Silence on the relevant literature and errors in implementation. *Nature Biotechnol.* **33**, 336–339 (2015).

[6] Weigt, M., White, R. A., Szurmant, H., Hoch, J. A. & Hwa, T. Identification of direct residue contacts in protein-protein interaction by message passing. *Proc. Natl. Acad. Sci.* **106**, 67–72 (2009).

[7] Kholodenko, B. N. *et al.* Untangling the wires: a strategy to trace functional interactions in signaling and gene networks. *Proc. Natl. Acad. Sci.* **99**, 12841–12846 (2002).

[8] Kemmeren, P. *et al.* Large-Scale Genetic Perturbations Reveal Regulatory Networks and an Abundance of Gene-Specific Repressors. *Cell* **157**, 740–752 (2014).

[9] Giaever, G. *et al.* Functional profiling of the Saccharomyces cerevisiae genome. *Nature* **418**, 387–391 (2002).

[10] Schaffter, T., Marbach, D. & Floreano, D. GeneNetWeaver: in silico benchmark generation and performance profiling of network inference methods. *Bioinformatics* **27**, 2263–2270 (2011).

[11] Prill, R. J. *et al.* Towards a rigorous assessment of systems biology models: The DREAM3 challenges. *PLOS ONE* **5**, e9202 (2010).

[12] Benjamini, Y. & Hochberg, Y. Controlling the false discovery rate: a practical and powerful approach to multiple testing. *J. R. Stat. Soc.* 289–300 (1995).

[13] Cherry, J. M. *et al.* Saccharomyces Genome Database: the genomics resource of budding yeast. *Nucleic Acids Res.* **40**, D700–D705 (2012).

[14] Siegenthaler, C. & Gunawan, R. Assessment of Network Inference Methods: How to Cope with an Underdetermined Problem. *PLOS ONE* **9**, e90481 (2014).

[15] Stolovitzky, G., Prill, R. J. & Califano, A. Lessons from the DREAM2 challenges - a community effort to assess biological network inference. *Ann. N.Y. Acad. Sci.* **1158**, 159–195 (2009).
