# Supplementary material for: Experimental noise cutoff boosts inferability of transcriptional networks in large-scale gene-deletion studies
Source: Nat Commun. 2018 Jan 9;9:133. doi: 10.1038/s41467-017-02489-x (PMC5760630; doi:10.1038/s41467-017-02489-x)
Supplement: Supplementary file 3 — Description of Additional Supplementary Files [file 41467_2017_2489_MOESM3_ESM.pdf]

Description of Additional Supplementary Files

File Name: Supplementary Data 1
Description: Table of links inferred from the yeast deletome data set using a stringent cutoff for the selection of significantly affected genes.

File Name: Supplementary Data 2
Description: Table of links inferred from the yeast deletome data set using a moderate cutoff for the selection of significantly affected genes.

File Name: Supplementary Data 3
Description: Table of hub nodes inferred from the yeast deletome data set.

File Name: Supplementary Data 4
Description: Table with overall scores as well as AUROC and AUPR values for all networks from the DREAM3 in silico network inference challenge.
